# Time Burden in Patients With Metastatic Breast and Ovarian Cancer from Clinic and Home Demands

**DOI:** 10.1001/jamanetworkopen.2025.49957

**Published:** 2025-12-16

**Authors:** Rachel I. Vogel, Patricia Jewett, Helen Parsons, Katherine Brown, Alyssa Pecoraro, Indya Starks, Stacey Ingram, Zuofu Huang, Fibiana Oladipo, Arjun Gupta, Deanna Teoh, Yingling Fan, Anne Blaes, Rebecca Arend, Gabrielle Rocque, Julian Wolfson

**Affiliations:** 1 Obstetrics, Gynecology and Women’s Health, University of Minnesota, Minneapolis; 2 Masonic Cancer Center, University of Minnesota, Minneapolis; 3 Environmental Health Sciences, University of Minnesota, Minneapolis; 4 Division of Health Policy and Management, University of Minnesota, Minneapolis; 5 Divisions of Hematology and Oncology and Gerontology, Geriatrics, and Palliative Care, University of Alabama at Birmingham O’Neal Comprehensive Cancer Center, Birmingham; 6 Division of Biostatistics and Health Data Science, University of Minnesota, Minneapolis; 7 Division of Gynecologic Oncology, University of Alabama at Birmingham, O’Neal Comprehensive Cancer Center, Birmingham; 8 Division of Hematology, Oncology and Transplantation, University of Minnesota, Minneapolis; 9 Humphrey School of Public Affairs, University of Minnesota, Minneapolis; 10 O’Neal Comprehensive Cancer Center, University of Alabama at Birmingham, Birmingham

## Abstract

This cohort study evaluates time demands among patients with metastatic breast and ovarian cancer.

## Introduction

Most studies on cancer-related time burdens rely on administrative or medical record data, failing to accurately measure time spent on traveling or waiting, home care tasks, scheduling, and paperwork.^1,2,3^ We used a previously tested smartphone app to capture time demands among patients with advanced cancer, a population at high risk of time burdens.

## Methods

We conducted a longitudinal cohort study of adults with metastatic breast or advanced-stage ovarian cancer to examine daily time use and cancer-related care burdens. The protocol was approved by the University of Minnesota institutional review board and registered with ClinicalTrials.gov (NCT05708703). All participants provided written informed consent. Study methods and results are presented in accordance with Strengthening the Reporting of Observational Studies in Epidemiology (STROBE) reporting guidelines. Expanded methods are provided in eMethods in Supplement 1.

Eligible individuals receiving systemic therapy for metastatic breast or advanced-stage ovarian cancer were recruited December 2023 to September 2024 at the University of Minnesota and University of Alabama at Birmingham. Participants completed baseline and follow-up surveys and used a mobile app, Daynamica, to track time use for 28 days. The app uses GPS and phone sensor data to automatically infer a user’s location and activity type. Each day is segmented into alternating activities (location-based episodes) and trips (movement between locations). At the end of each day, participants are asked to review, correct, and label any unlabeled activities, provide additional details about facility-based cancer care episodes, and identify any nonfacility-based (eg, at-home) cancer care activities they engaged in via daily app-based surveys (eFigure in Supplement 1). Analyses focused on participants with sufficient app engagement (≥7 days). Data were summarized using descriptive statistics.

## Results

Among the 60 participants (median [range] age 59 [30-78] years), 11 (18%) self-identified as Black, 46 (77%) as White, and 2 (3%) as more than 1 race (Table). At baseline, 17 (28%) were receiving initial treatment, 9 (15%) receiving maintenance treatment, and 34 (57%) receiving therapy for recurrence or progression. Participants who provided sufficient app data were similar to those who did not.

**Table.** Participant Demographic and Clinical Characteristics Among All Participants and Among Those Who Engaged With the Mobile App at Least 7 Days

Characteristic	Participants, No. (%)	
	All (N = 78)	≥7 Days’ app engagement (n = 60)
Age, median (range), y	59 (23-78)	59 (30-78)
Time since diagnosis, median (range), y	1.8 (0.1-11.0)	1.7 (0.1-11.0)
Out-of-home health care days in electronic medical record within 28 d of study entry, median (range), d	4 (1-10)	4 (1-10)
Self-reported race		
Black	15 (19)	11 (18)
More than 1 race	2 (3)	1 (2)
White	59 (76)	46 (77)
Other^a^	2 (3)	2 (3)
Self-reported ethnicity		
Hispanic	4 (5)	4 (7)
Non-Hispanic	71 (91)	54 (90)
Unknown	3 (4)	2 (3)
Employment status		
Full time	26 (33)	20 (33)
Part time	5 (6)	5 (8)
Not working	44 (56)	35 (58)
Missing	3 (4)	0
Education		
High school graduate	14 (18)	11 (18)
Vocational/associate’s degree	8 (10)	7 (12)
Some college	14 (18)	11 (18)
College graduate	19 (24)	13 (22)
Graduate or professional training	23 (30)	18 (30)
Dependents		
No	29 (63)	39 (65)
Yes	49 (37)	21 (35)
Annual household income, US $		
<50 000	26 (33)	19 (32)
50 000-99 999	22 (28)	16 (27)
100 000-149 999	8 (10)	7 (12)
≥150 000	15 (19)	12 (20)
Prefer not to answer	7 (9)	6 (10)
Cancer clinic		
University of Alabama, Birmingham	40 (51)	29 (48)
University of Minnesota	38 (49)	31 (52)
Distance from the clinic, miles		
<30	38 (49)	30 (50)
≥30	40 (51)	30 (50)
Cancer type		
Metastatic breast cancer	39 (50)	32 (53)
Advanced stage ovarian cancer	39 (50)	28 (47)
Treatment status		
Front-line/up-front treatment	23 (30)	17 (28)
Maintenance	10 (13)	9 (15)
Treatment for recurrent/progression disease	45 (58)	34 (57)

^a^ Self-reported race as Hispanic (1), Latino (1).

Participants reported a mean (SD) of 4.2 (2.6) out-of-home cancer-related care episodes (eg, clinic, hospital, or pharmacy) over 28 days. Episodes most often involved treatment (113 episodes [36%]), clinic visits (94 episodes [30%]), and/or laboratories (100 episodes [31%]). A total of 141 episodes (44%) had reported wait times less than 15 minutes, 61 (19%) had no wait, and 46 (14%) had waits more than 60 minutes. Median (IQR) travel time per episode was 35 (20-57) minutes; the time spent traveling and waiting for care often exceeded time spent receiving care.

Participants engaged in at least 1 at-home cancer-related task on 1557 study days (80%), with a median (IQR) of 209 (121-355) minutes per week spent taking medications, scheduling appointments, handling medical bills, managing symptoms, monitoring health status, seeking information about cancer, and arranging help or transportation. In total, participants spent a median (IQR) of 400 (266-603) minutes per week on cancer-related tasks (Figure). Over one-third (21 participants [35%]) said cancer-related tasks disrupted daily activities, including self-care, chores, work, or socializing, on more than half of their days.

**Figure.**
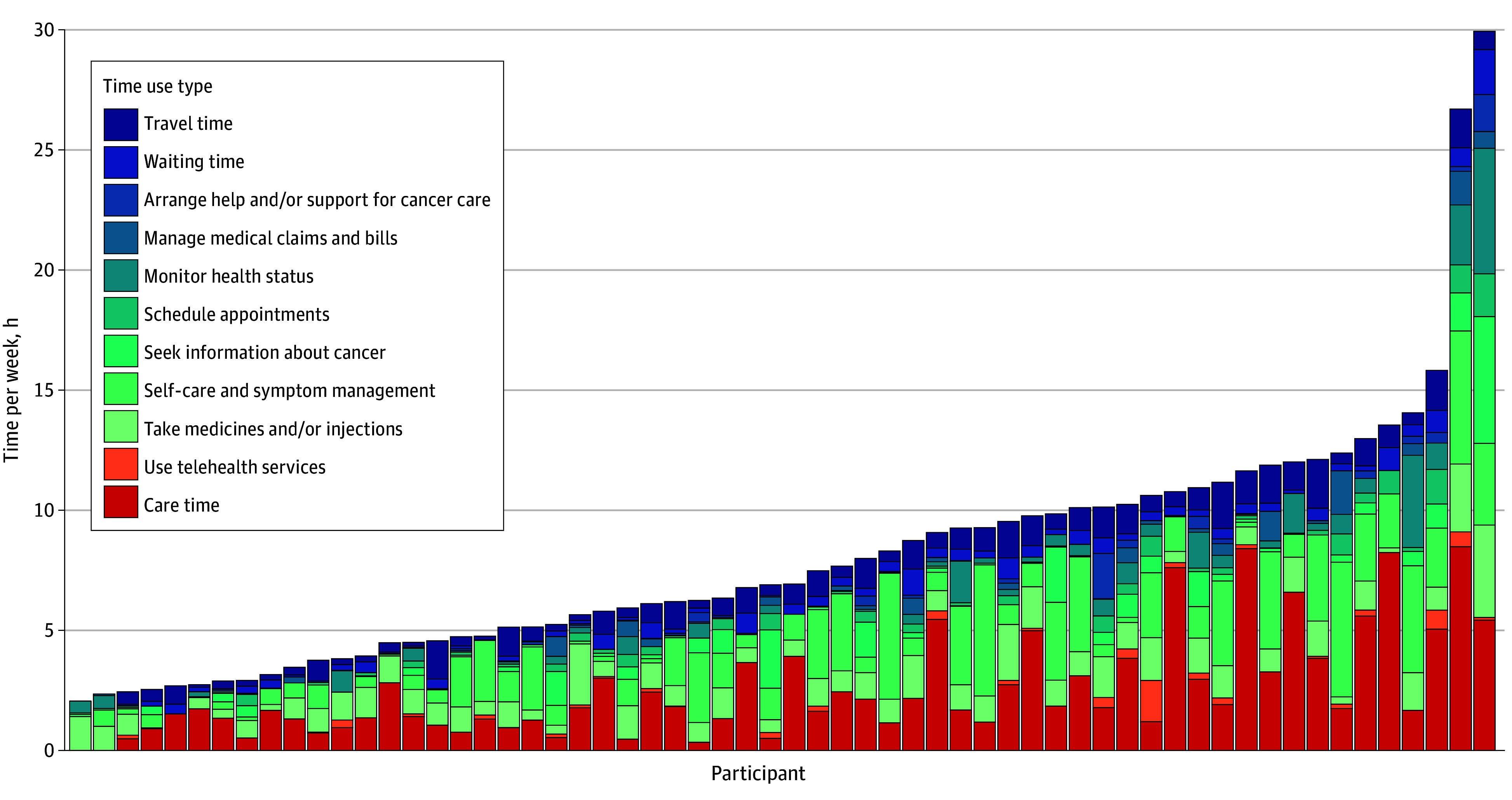
Weekly Total Time Spent on Each Health Care, Travel Waiting, and Home Cancer-Related Task by Participant Each column represents an individual participant. Care time included all time spent out of home at a medical facility and labeled as cancer care; travel and waiting time were associated with these cancer care visits. All other types (taking medications or injections, self care and symptom management, scheduling appointments, monitoring health status, managing medical claims and bills, and arranging help or support for cancer care) were reported in the end-of-day survey as occurring outside of a health care facility.

## Discussion

Using the app, we captured detailed time use data among individuals with advanced ovarian and metastatic breast cancer receiving treatment. Half of participants spent approximately 7 hours per week on cancer-related tasks, with most reporting cancer-related tasks daily. Participants reported spending about 2 hours on home care and 1 hour on travel or waiting for every hour of direct care, though time demands varied widely. Our findings align with previous estimates that older cancer survivors interact with the health care system on average 1 day per week^4^ and advanced stage survivors on 20% of all days.^5,6^

Limitations include the pilot design, exclusion of non-English speakers, and a sample restricted to women with advanced ovarian or metastatic breast cancer. The 28-day data collection window prevented capturing time burdens near diagnosis or how they evolve over longer periods. Future work should refine time burden measures by understanding how participants report each care component. Next steps include examining demographic and clinical factors linked to time burdens and quantifying their impact on patient and caregiver quality of life, employment, and financial outcomes, with the end goal of reducing time burden through patient-centered interventions.

## Notes

Supplement 1. eMethods. eFigure. Screenshots of Daynamica App

Supplement 2. Data Sharing Statement

## References

[1] Yabroff KR, Guy GP Jr, Ekwueme DU, . Annual patient time costs associated with medical care among cancer survivors in the United States. Med Care. 2014;52(7):594-601. doi:10.1097/MLR.0000000000000151 24926706 PMC4058637

[2] Rocque GB, Williams CP, Ingram SA, . Health care-related time costs in patients with metastatic breast cancer. Cancer Med. 2020;9(22):8423-8431. doi:10.1002/cam4.3461 32955793 PMC7666754

[3] Kagalwalla S, Tsai AK, George M, . Consuming patients’ days: time spent on ambulatory appointments by people with cancer. Oncologist. 2024;29(5):400-406. doi:10.1093/oncolo/oyae016 38339991 PMC11067814

[4] Gupta A, Chant ED, Mohile S, . Health care contact days among older cancer survivors. JCO Oncol Pract. 2024;20(7):943-952. doi:10.1200/OP.23.00590 38452315 PMC11268556

[5] Gupta A, Nguyen P, Kain D, . Trajectories of health care contact days for patients with stage IV non-small cell lung cancer. JAMA Netw Open. 2024;7(4):e244278. doi:10.1001/jamanetworkopen.2024.4278 38587847 PMC11002696

[6] Ording AG, Skjoth F, Poulsen LO, . Time toxicity of systemic anticancer therapy for metastatic lung cancer in routine clinical practice: a nationwide cohort study. JCO Oncol Pract. 2024;OP2400526. doi:10.1200/OP-24-00526 39705615

