# Supplementary material for: Time Burden in Patients With Metastatic Breast and Ovarian Cancer from Clinic and Home Demands
Source: JAMA Netw Open. 2025 Dec 16;8(12):e2549957. doi: 10.1001/jamanetworkopen.2025.49957 (PMC12709373; doi:10.1001/jamanetworkopen.2025.49957)
Supplement: Supplement 1. — eMethods. eFigure. Screenshots of Daynamica App [file jamanetwopen-e2549957-s001.pdf]

Supplemental Online Content

Vogel RI, Jewett P, Parsons H, et al. Time burden in patients with metastatic breast and ovarian cancer from clinic and home demands. *JAMA Netw Open*. 2025;8(12):e2549957. doi:10.1001/jamanetworkopen.2025.49957

eMethods.

eFigure. Screenshots of Daynamica App

This supplemental material has been provided by the authors to give readers additional information about their work.

© 2025 Vogel RI et al. *JAMA Network Open*.

eMethods

Study Design

We conducted a longitudinal study of individuals diagnosed with and currently receiving treatment for metastatic breast cancer or advanced stage (stage III or IV) ovarian cancer (including fallopian tube and primary peritoneal carcinoma).

Participants were asked to complete a survey at study entry (baseline survey), download and utilize the mobile application Daynamica (details below) to track daily time use for 28 days (sensor data + daily surveys), and a final survey following the daily data collection period (follow-up survey). Participants were compensated up to $175 for completing data collection. This study protocol was approved by the University of Minnesota Institutional Review Board (STUDY00016834) and registered with ClinicalTrials.gov (NCT05708703).

Study Population and Recruitment

Participants were recruited through cancer clinics within the University of Minnesota MHealth Fairview system and the University of Alabama at Birmingham between December 2023 and September 2024. Potentially eligible patients were approached at the time of a scheduled clinic visit by dedicated research staff in collaboration with their healthcare provider. We sought to enroll 80 individuals (~20 of each cancer type at each site), replacing individuals who did not complete the baseline survey and initiate mobile app data collection. Our planned sample size of 80 participants struck a balance between feasibility and statistical precision and power to estimate the quantities of interest. For descriptive statistics of individual-level measures (e.g., mean number of minutes per week spent on cancer care related activities) on the entire sample, we will be able to estimate means of continuous measures with a margin of error of ±0.22 standard deviations. For comparing means of individual-level measures between two equal-sized subgroups (e.g., those with breast and ovarian cancer), our sample size yields 80% power to detect a difference of 0.64 standard deviations.

© 2025 Vogel RI et al. *JAMA Network Open*.

The inclusion criteria included age greater than 18 years, a diagnosis of metastatic (stage IV) breast cancer or advanced stage (stage III or IV) ovarian, primary peritoneal, or fallopian tube cancer, currently receiving systemic anti-cancer therapy, and ability to read and write English. Individuals were not required to own a smartphone and could receive one from the study team for the duration of the study if needed. Participants were eligible regardless of time since original diagnosis. Exclusion criteria included individuals who were unable to provide informed consent, in prison, or in hospice care. All participants provided in-person paper or electronic written informed consent utilizing Research Electronic Data Capture (REDCap) eConsent[5] before performing any study related procedures.

Study Procedures and Data Collection

Following consent and completion of the baseline survey, participants were provided instructions on how to download the Daynamica mobile application and a demonstration on how to use it. Daynamica is an established, easy-to-use sensor-based tool to collect data automatically on daily activities and time use requiring minimal user input. The app has been used in prior research studies; we utilized a customized version of the publicly available mobile application which included study-specific daily surveys and routed all study data to a secure, HIPAA-compliant University of Minnesota server.

For this study, participants were asked to install the Daynamica app on their smartphones for a 28-day period. The Daynamica app relies primarily on GPS location data paired with information from other phone sensors (e.g., accelerometer, gyroscope) to infer a user's location and activity type. At the most fundamental level, the app automatically separates a user's day into distinct episodes which are of two alternating types: activities, which take place at a specific location, and trips, during which individuals move between activities. Mode of transport for trips (car, walk, bike, etc.) is automatically sensed using smartphone data. Activity type is inferred based on location labels provided by users; for example, once a user labels an activity taking place at a given location as "Home", then the app will automatically apply this label to all future activities taking place at that location. While engaged in active data collection, patients using the app are asked to review, correct, and label

© 2025 Vogel RI et al. *JAMA Network Open*.

any unlabeled episodes at the end of each day; they are also asked to provide additional details about any facility-based cancer care episodes, to identify any non-facility-based cancer care activities that they engaged in, and to self-report their distress level. The app captures location information and automatically infers time, type, and location of activities and trips, providing a "calendar view" of each day (**eFigure 1a**). Participants were asked to label and provide additional details about each activity or trip (“episode”, **eFigure 1b**), with episodes noted as being cancer-related care prompting participants to further denote the type of care received (selecting all that apply): labs, treatment, clinic visit, research, pharmacy, imaging, other, and to estimate time spent waiting during that episode (no wait, <15 min, 15-30 min, 30-60 min, >60 min; **eFigure 1c**). If Daynamica users repeatedly visited the same care location, these episodes were automatically recognized and labeled. At the end of each day, participants were asked to complete a short (<5 minute) survey, including questions regarding any cancer-related phone calls or activities during that day that were not otherwise reported, including time spent on those activities. Lastly, participants were asked daily to report their distress level on 0-10 Likert scale[11] (**eFigure 1d**). Daily surveys were required to be fully completed to be submitted.

eFigure. Screenshots of Daynamica App

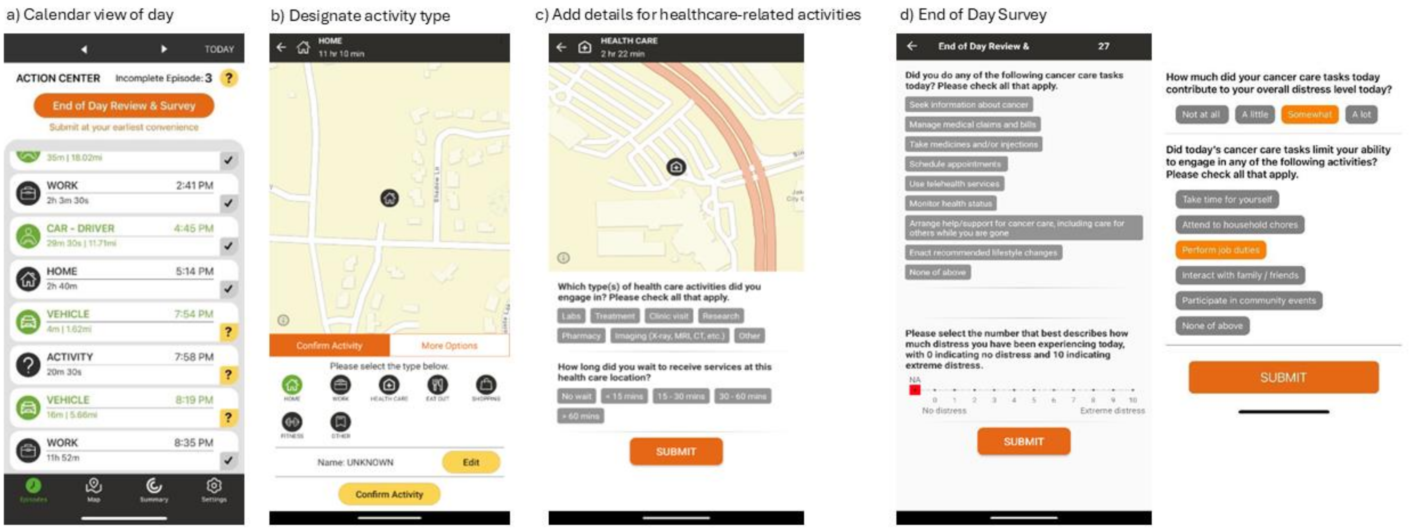

Participant demographics were self-reported at baseline, and patient-reported outcomes, including general quality of life, depression, anxiety, and financial concerns, were collected at baseline and follow-up (after the 28

© 2025 Vogel RI et al. *JAMA Network Open*.

days of mobile application data collection). Demographics collected included age, race/ethnicity, household income, education, insurance status, relationship status, parenting and caregiver status, and employment status. Surveys were administered on paper or electronically in REDCap per participant preference. Details regarding participant's cancer diagnosis and medical history, including diagnosis, treatments received, adverse events, comorbidities, and healthcare encounters, were abstracted from the medical records at each site and entered into REDCap.

Data Processing and Statistical Analysis

After data collection, the following data processing steps were performed to ensure data completeness and usability. First, only data from participants who meaningfully participated in the study were analyzed. To this end, participants were only included in the data analysis if they had completed at least seven days of end-of-day survey and seven days of activity data collection, although partial missingness of activity data was allowed throughout the day. We defined a healthcare episode as a continuous period of five or more minutes spent at the same location which was labeled as an out-of-home healthcare interaction by a participant. Participant demographic and clinical characteristics were subsequently summarized using descriptive statistics. Plots and tables were generated by combining episodic data and end-of-day surveys.

A total of 100 potentially eligible individuals were approached, 89 (89%) enrolled and provided consent, and 80 completed the baseline survey. Two participants withdrew from the study, 12 did not collect any mobile app activity data, three did not provide at least seven days of activity data, and three did not complete at least seven mobile app end-of-day surveys, resulting in a final analytic sample size of 60 (75% of enrolled participants, 60% of those approached). Of those, 52 (87%) provided at least 21 days of mobile app data and 43 (72%) provided 28 days or more.

To examine the accuracy of the we compared the days with healthcare episodes reported in the Daynamica app with information on days interacting with the healthcare system available in the electronic medical record

© 2025 Vogel RI et al. *JAMA Network Open*.

(EMR). On average, we detected more days interacting with the healthcare system in Daynamica (median: 4, Q1-Q3: 2-6) than from the EMR (median: 3, Q1-Q3: 2-4). Just under half (41%) had more days reported using the Daynamica app, even after excluding pharmacy only days which were captured by the app and not in the EMR; for the 34% with fewer episodes captured by the Daynamica app than the EMR, the vast majority were missing one day, typically the day of enrollment (i.e. were recruited at a clinic appointment / treatment and that day was not captured by the app).

© 2025 Vogel RI et al. *JAMA Network Open*.
