# Supplementary material for: Time Burden in Patients With Metastatic Breast and Ovarian Cancer from Clinic and Home Demands
Source: JAMA Netw Open. 2025 Dec 16;8(12):e2549957. doi: 10.1001/jamanetworkopen.2025.49957 (PMC12709373; doi:10.1001/jamanetworkopen.2025.49957)
Supplement: Supplement 2. — Data Sharing Statement [file jamanetwopen-e2549957-s002.pdf]

Data Sharing Statement

Vogel. Time Burden in Patients With Metastatic Breast and Ovarian Cancer from Clinic and Home Demands. *JAMA Netw Open*. Published December 16, 2025. doi:10.1001/jamanetworkopen.2025.49957

Data
Data available: Yes
Data types: Deidentified participant data, Data dictionary
How to access data: De-identified demographic data collected from the baseline survey are publicly available (https://doi.org/10.13020/BWYR-QK74); due to privacy restrictions other data may be available upon request to the corresponding author and review by the University of Minnesota Institutional Review Board.
When available: With publication

Supporting Documents
Document types: None

Additional Information
Who can access the data: Deidentified survey data will be available to anyone requesting the data.
Types of analyses: Any purpose
Mechanisms of data availability: Publically available
